# The ‘excess gas’ method for laboratory formation of methane hydrate-bearing sand: geotechnical application

**DOI:** 10.1038/s41598-021-00777-7

**Published:** 2021-11-11

**Authors:** Lior Rake, Shmulik Pinkert

**Affiliations:** Civil and Environmental Engineering Department, Faculty of Engineering Sciences, Ben-Gurion University of the Negev, Beer Sheva, Israel

**Keywords:** Civil engineering, Energy science and technology

## Abstract

Over recent years, there has been a growing interest in producing methane gas from hydrate-bearing sands (MHBS) located below the permafrost in arctic regions and offshore within continental margins. Geotechnical stability of production wellbores is one of the significant challenges during the gas extraction process. The vast majority of geotechnical investigations of MHBS have been conducted on laboratory-formed samples due to the complex procedure of undisturbed sample extraction. One of the most commonly used hydrate laboratory-formation methods is the excess-gas method. This work investigates fundamental aspects in the excess-gas formation of MHBS that are affecting the geotechnical interpretation and modeling. The work finds that (1) the measured temperature in the experimental system may be quite different from the in-sample temperature, and can reach 4 $^\circ$C difference during thermodynamic processes. This potential difference must be considered in investigation of hydrate formation or dissociation, (2) various calculation approaches may yield different hydrate saturation values of up to tens of percentages difference in high hydrate saturations. The calculation formulas are specified together with the fundamental difference between them, (3) the water mixture method during the sample assembling is critical for homogeneous MHBS laboratory formation, in which a maximum initial water content threshold of 9.1 to 1.3 % are obtained for a minimal fraction size of 0.01 to 0.8 mm, respectively, (4) the hydrate formation duration may influence the MHBS properties, and should be rigorously estimated according to the real-time gas consumption convergence. The outcomes of this work may contribute to the integration of data sets derived from various experiments for the study of MHBS mechanical behavior.

## Introduction

Methane hydrate is a solid crystalline cage of water molecules encapsulating a methane gas molecule^1^. The hydrate structure is stable under thermodynamic conditions of high pressure and low temperature, which can be typically found in marine sediments of continental shelves and under the permafrost^2^. The vast abundance of methane hydrate worldwide attracts the interest of energy production corporations and researchers. In the gas production process, the gas is dissociated from the hydrate-bearing soil by inferring the hydrate phase conditions. The dissociation process involves mechanical effects that may cause a wellbore instability, and therefore should be carefully studied and simulated^3,4^. Gas production from hydrate-bearing sands (MHBS), rather than from fine-grain soils, is considered more efficient for production due to the relative high permeability of sands. The common geotechnical investigation methods for MHBS use sediment bulk definitions, such as strength, stiffness, and dilation, which have been laboratory evaluated over the past few decades e.g.,^2,5–14^. The MHBS bulk properties are studied through geotechnical testing, in which the vast majority of the experiments has been conducted on laboratory formed samples, as an alternative to the complicated and expensive natural MHBS extraction^15^. The use of artificial MHBS in mechanical testing also enables a repeatable, and thus more reliable, experimental process. However, the pore-space distribution (morphology) of hydrate may dramatically vary among MHBS associated with different hydrate formation methods.

One can divide the laboratory hydrate formation methods into three main categories:

1. *Excess Water Method*, in which the soil is subjected to a limited amount of methane gas while distilled water is freely injected into the sample to produce the desirable pore pressure. In this method, the amount of hydrate is governed by the gas volume. The hydrate may constitute a part of the sediment load-bearing system, depending on the hydrate saturation level^10^.
2. *Dissolved Gas Method*, in which the sample is continuously circulated with water dissolved methane-gas under the hydrate thermodynamic stability conditions. The hydrate, in this method, crystallized from the grain surface into the pore space, in a non-homogeneous morphology which depends on the water percolation directions^2,16–19^. The hydrate saturation is governed by the duration of the circulation process and may be limited by flow blockages.
3. *Excess Gas Method*, in which the hydrate is crystallized from limited water or ice amount in the sample while free methane gas is provided. In this method, the initial pore water distribution in the soil pore-space dominates the hydrate morphology. When the hydrate is formed from capillary water, it would crystallize at the pore-space grain-contacts, while when the hydrate is formed from ice-seeds, the hydrate would crystallize inside the pores and may be part of the sediment load-bearing system, depending on the ice amount^2^.

Note, that a combined method can be employed, such as initiating an excess-gas formation followed by a dissociation-reformation process, yielding a pore-filling morphology^20^. Each of the formation methods produces a different hydrate morphology, which affects the MHBS mechanical response differently.

Hydrate formation in soil samples is time-consuming, which that can be lasting hours to weeks. The entire testing process is considered complex, as it characterized by multi-system functioning with high accuracy demands; for example, small differences between high cell-pressure and pore-pressures, sensitive gas volume measurements at a varied temperature environment, thin and flexible membranes to hold a high-pressure difference, and numerous electric and mechanical components with continuous functioning throughout the entire process. For these reasons, most of the acquired experimental knowledge on MHBS, which stands at the basis of most of the developed mechanical models, is based on MHBS formed using the excess-gas method, which is considered less complicated and conceivably repeatable with comparison to the other methods^5,7,10,21–29^. Still, quite a few differences in testing procedures and results’ analysis can be found among these works, in aspects such as (1) *hydrate saturation calculation*, where some works use gas conversion of either the collected gas during dissociation or the consumed gas during the hydrate formation process, while other works calculate a mass conversion of the initial water content. Besides, not all works report their hydrate-saturation calculation scheme; (2) *formation time*, where the duration of hydrate formation is varied among the different works, in which the start-point and end-point criteria of the formation process are not consistently reported; (3) *sample preparation*, where the partially water-saturation state of the assembled sand can be achieved either by using moist sand, sand mixed with ice-powder or by draining excess water from a saturated sample. More importantly, homogeneity verification of the partly saturated sample during the preparation stage is commonly not reported. This verification is important because non-uniform water distribution yields non-homogeneous hydrate distribution; (4) *water saturation*, in which some works use the process of water saturation after hydrate formation, replacing the remaining gas in the sample. This process is not standard, as it may be associated with hydrate blockages and local dissociation/reformation issues.

In this paper, these laboratory aspects are thoroughly examined by experimental investigation. It should be noted that the authors do not intend to question the validity of previous works, but rather to refine their experimental outcomes to improve the integration of different experimental results in mechanical analysis.

## Experimental system and procedures

**Figure 1:**
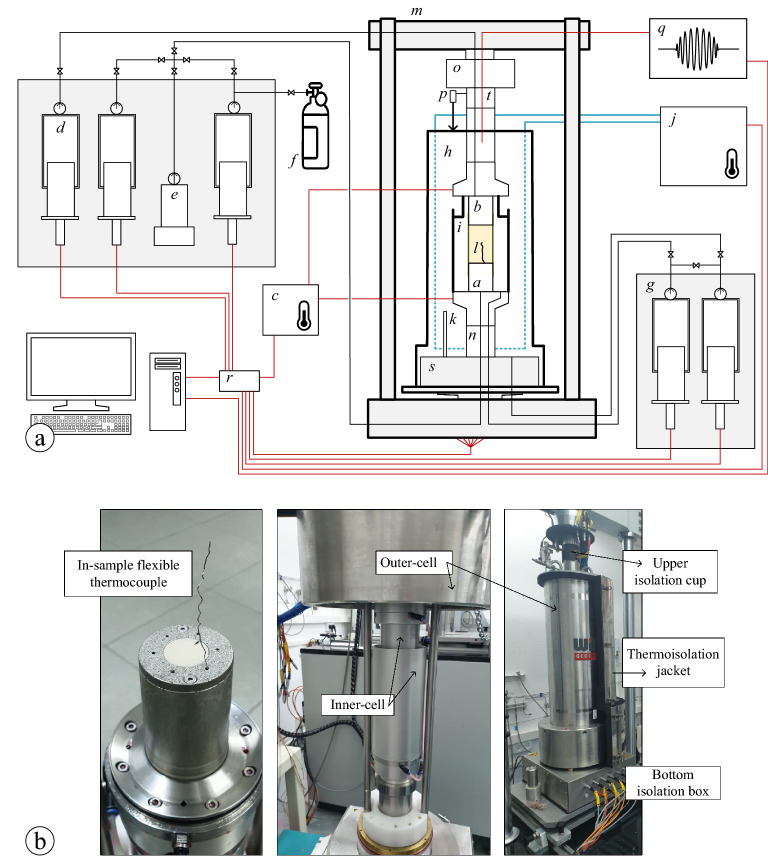
(**a**) An itemized illustration of the experimental setup used in this work (**b**) and photos of selected parts of the system.

The vast majority of the MHBS mechanical investigations have been carried out using designated triaxial testing systems for MHBS. These systems enable controlling and monitoring the thermodynamic conditions (pressure and temperature), stress differences (between vertical and horizontal stresses, and between the sample pore-pressure and the applied cell-pressure), and the sample deformations (vertical, radial, and volumetric change). The testing apparatus is composed of various sub-systems and components, which may vary among the different laboratories by their mechanical capacities, technical specification, the degree of accuracy, and the real-time servo-control method. The experimental study in this work was conducted using a state-of-the-art experimental triaxial test system for MHBS investigation, which is described in Fig. 1, and detailed in the list below.

### Triaxial test apparatus for MHBS

In the test procedure, a cylindrical soil sample in 50/100 mm diameter/height is assembled between (a) bottom and (b) top caps that include ultrasonic (pressure and shear) wave transducers and are heat controlled through (c) an electrical heating system. The sample is wrapped in a latex membrane, separating between the sample pore-pressure and the external cell-pressure fluids. The pore-pressure is obtained either by gas or liquid pressure which are generated through a unique pressure/volume controller system which includes (d) three 600 ml automatic piston pumps of 20 MPa pressure capacity with 0.05% accuracy. Two of the pumps are connected to (e) a water-methane mixing vessel for injection of dissolved-gas water into the sample. One of the piston pumps is connected to (f) an external gas supply, and all the three pumps can be connected to the sample through the bottom and top cups, equally. The external pressure is generated through a unique double-wall cell system, in which (g) a pair of 300 ml piston pumps are pressurizing both (h) an outer stainless-steel cell and (i) a relatively small inner cell, where the entire system has a pressure capacity of 25 MPa with 0.05% accuracy. The inner cell is composed of two pieces separated by a low-friction interface, in which the lower and upper pieces are fixed on the bottom and top cups, respectively, to enable the inner-cell volume change. The double-wall cell system is designed for accurate sample volume change measurements, during the test, by the volume change response of the inner-cell pump. The temperature of the entire system is dictated by the cell-liquid temperature, which is controlled by inner circumferential lines that circulate cooling liquid at a required temperature ($-15^\circ \hbox {C}$ to $+40^\circ \hbox {C}$ range) through a circulating pump that connected to (j) a temperature control system ($0.1^\circ \hbox {C}$ accuracy). The target temperature is achieved by a closed-loop servo control system that includes thermometers located at (k) the outer cell, the bottom and top cups, and on top of (l) a flexible wire running through the sample. The cell is located in (m) a load-frame with a 100 kN capacity, which enables closed-loop control of displacement and force (or strain and stresses, respectively). The vertical force is measured using (n) a submersible load cell with 100 kN capacity (0.1% accuracy), which can be calibrated and verified by an additional (o) outer load cell. The vertical displacement of the sample is monitored by both the lifting piston gauge and (p) an external linear displacement transducer. Throughout the whole test, automatic P and S wave signals are sent and recorded using (q) an ultrasonic wave measuring system. In addition, the system can be adapted to accommodate 70/140 mm diameter/height samples, but without the double-wall cell system. In this case, an internal vertical and circumferential strain measurement kit can be used for volume change measurements. All the servo-controlled sub-systems are connected to the computing system using network cables (marked by red color in the figure) through (r) a communication network switch. For the system thermal isolation, the cell is separated from the working table by (s) a bottom thermoisolation box and (t) an upper thermoisolation cup, through which all the system cables and pipes are safely passing, in addition to isolation jackets covering both the cell sideways and top. Figure 1b presents different views of selected parts in the system.

### Testing procedure

This section describes the procedures of MHBS production (using the excess-gas method) and triaxial testing employed in this work. Frozen sand is mixed with an ice-powder to create homogeneous moist sand with the desired moisture content, and compacted into 50 mm diameter mold in ten layers. The top cap is installed, and the sample is vacuumed before the mold removal. The inner and the outer cells are installed, filled up with the confining liquid, and stabilized at 100 kPa. Both the inner and outer cells are pressurized by 9 MPa at a rate of 100–200 kPa/min, simultaneously with the back-pressure (gas) increase to 8 MPa by injecting methane gas into the sample, while maintaining a 800–1000 kPa pressure difference (i.e., effective pressure). Then, the cell-liquid temperature is reduced to $3\,^\circ \hbox {C}$, while measuring the temperature change by four thermometers located in the cell liquid, in the top and bottom caps, and within the soil sample. The system is kept stable over more than 72 hours, during which the methane-hydrate is crystallized. During the hydrate formation, the gas consumption (by the hydrate) is measured, and ultrasonic P and S wave signals are monitored. After the MHBS formation, the sample are vertically loaded under drained conditions at a rate of 0.1 %/min, until the sample reaches failure. During the loading process, the axial deformation, the volume change of the sample, and the axial force are monitored. At the end of the mechanical loading, the hydrate is dissociated under undrained conditions (constant volume), while keeping a constant effective pressure by a real-time update of the cell pressure (as a response to the gas pressure increase in the sample). Finally, the methane gas is released and measured.

## Accurate temperature measurements

Hydrate stability depends on maintaining its phase boundary conditions of pressure and temperature. The pressure control can be achieved directly, but the sample temperature has to be regulated in an indirect and time-dependent manner. The thermal conductivity of the sample and the test apparatus plays a significant role in controlling and monitoring the sample temperature. Temperature regulation occurs through real-time thermometer responses, which their position within the system may be essential. In static MHBS testing, where no deformation is expected, the thermometer may be placed inside the sample e.g.,^20,25,30^. However, in a case where the MHBS experiences mechanical changes, the thermometer stiffness may affect the mechanical response. Therefore, in mechanical testing of MHBS (such as triaxial test, consolidation, or direct shear) it is common to measure the temperature of the cell liquid, outside the sample e.g.,^7,10,21,23,29,31–34^.

**Figure 2:**
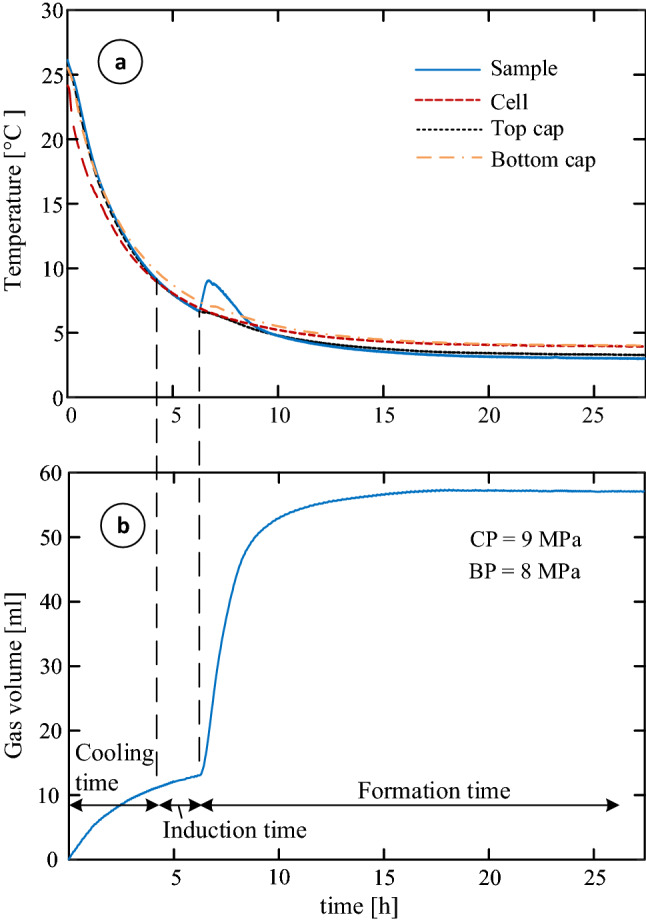
(**a**) Temperatures measured by the four thermometers in the system and (**b**) the gas consumption during the hydrate formation process.

This work examines the accuracy of temperature measurements outside the sample (which are commonly used), with comparison to in-sample measurements. To this end, four thermometers are placed in the experimental system; a resistance temperature detector (RTD) within the cell confining liquid (Fig. 1*k*), two resistance temperature detectors at the top and bottom cups (Fig. 1a,b), and a miniature thermocouple on top of a unique flexible wire at the middle of the sample (Fig. 1*l*). The flexible thermocouple wire allows temperature measurements during both the MHBS formation and the shearing process. Figure 2a shows a temperature comparison between the four thermometers in the system during the process of system cooling for the hydrate formation, under a cell pressure (CP) and a back pressure ($P_B$) of 9 and 8 MPa, respectively. Three clear trends can be observed from the figure; (1) *during initial cooling*, in which the temperature gradient is from the sample and outwards (i.e., the confining liquid is cooler than the sample), where a temperature difference of 3.5 $^\circ \hbox {C}$ was observed, (2) at the *final stage of hydrate formation*, during which the temperature gradient turns direction, as the sample reaches a stable - low - temperature while the outer cell is affected by the room temperature (even if great thermal isolation efforts are taken), where a temperature difference of $1^{\circ }\hbox {C}$ was observed, and (3) *at hydrate formation initiation*, in which the sample temperature spikes as the result of an exothermic reaction to the hydrate formation, where a temperature difference of $2.5^{\circ }\hbox {C}$ was observed. Similar temperature difference has been previously reported in the context of thermal gradients even inside the MHBS sample^35^.

Figure 2b shows the methane consumption during the sample cooling and the hydrate formation processes. It is apparent from the figure that the dramatic increase in gas consumption corresponded to the temperature spike (measured by the in-sample thermocouple; Fig. 2a). This measurement point, which was consistently observed in our measurements, marks the initiation of the detectable hydrate formation. The gas consumption rate at the beginning of the cooling process, before hydrate formation, is proportional to the temperature decrease, according to the ideal gas law. Note, that the sample reaches the methane-hydrate stability zone two hours before the detectable hydrate formation. This time-lag describes the initial time of nucleation, “where nucleation occurs on too small a size scale to be detected”^36^, which described in the figure as an ‘induction time’. In total, the observed formation time starts 6.2 hours after initiating the cooling process. These time-lags should be considered in the process of laboratory hydrate formation or in comparison between different data-sets from the literature.

**Figure 3:**
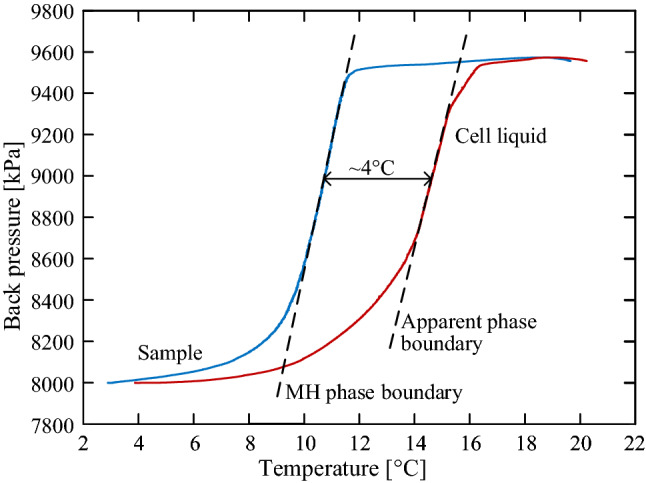
The thermodynamic response during the process of undrained thermal dissociation.

The difference between various temperature measurements can be also observed in the process of dissociation. Figure 3 shows temperature measurements of the in-sample thermocouple and the in-cell thermometer (which is the most commonly used in MHBS geotechnical testing systems). The described dissociation process is by increasing the temperature while keeping undrained conditions (i.e., volume-constrained gas-expansion). In this process, the $P_B$ increases as the thermodynamic conditions approaching the hydrate phase boundary and stabilized at the end of the dissociation. In accordance with the $P_B$ increase, the CP is real-time updated to keep a constant effective difference of 1 MPa between them. The endothermic response during the dissociation process is such that the pressure-temperature (PT) state tends to follow the phase boundary curve, as observed in previous studies^37–39^. As can be inferred from the figure, a noticeable difference of up to 4 $^\circ \hbox {C}$ is obtained between the in-sample and the in-cell thermometers. Different phase boundaries may be adjusted to the two measuring responses, in which the in-sample PT response follows the methane hydrate phase boundary while the in-cell PT response may be considered as an apparent phase boundary (the dashed curves in Fig. 3). Consideration of this significant temperature difference, which may arise in common geotechnical MHBS testing, is vital in simulations of thermodynamic processes such as dissociation/reformation.

## The effective duration of hydrate formation

The time duration of the hydrate formation process in the excess-gas method has been reported between the range of 17 hours to 3 days among the different works^5,7,10,21,23,26–28,30^, in which no consistent time-set criterion for the formation start and end points was determined. The deviation between the formation durations may be explained by the exponential decay in the hydrate formation progression, in which only a small portion of methane gas is consumed from the second day onwards. At the initial formation stage, the hydrate is formed rapidly at the water-gas interfaces and then the formation rate decays as the hydrate separates between the free gas and the remaining water^40^. Finally, even though the formation process was converging, it was still noted that not all water is converted into hydrate. This phenomenon may be due to a mechanism of trapped water inside hydrate shells^28,40^ or because of diffusion constraints that create thin water films between methane-hydrate and quartz^41^. Therefore, one should determine a reasonable standard for the hydrate formation duration, for which the formation process is effectively converged.

**Figure 4:**
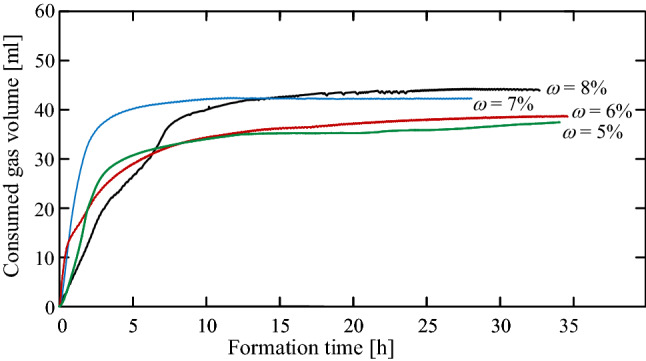
Consumed gas volume by hydrate crystallization during the formation process.

Figure 4 shows the net gas–volume consumed during the hydrate crystallization process in samples containing different initial water contents; $\omega = 5$, 6, 7, and 8%. The time axis shown in the figure starts at the exothermic reaction, which marks the formation initiation. Thermal gas-expansion effects are reduced in the presented curves, where the thermal expansion coefficients are quantified from the mechanical response to the cooling process (before the hydrate formation). Although the hydrate thermodynamic conditions were kept stable over 72 h, only the first 30–35 h are presented in the figure, during which the hydrate formation was converged. The figure shows an inconsistency in the gas consumption rate throughout the formation processes. After 17 h, the $\omega = 5$, 6, 7 and 8% samples achieved 94.3, 100, 94.2 and 97.5% of the total gas consumption, after 24 hours they consumed 96, 100, 97.9 and 99.5% of the gas, in which a full convergence was achieved after 35, 32, 11.5 and 27 hours, respectively.

**Figure 5:**
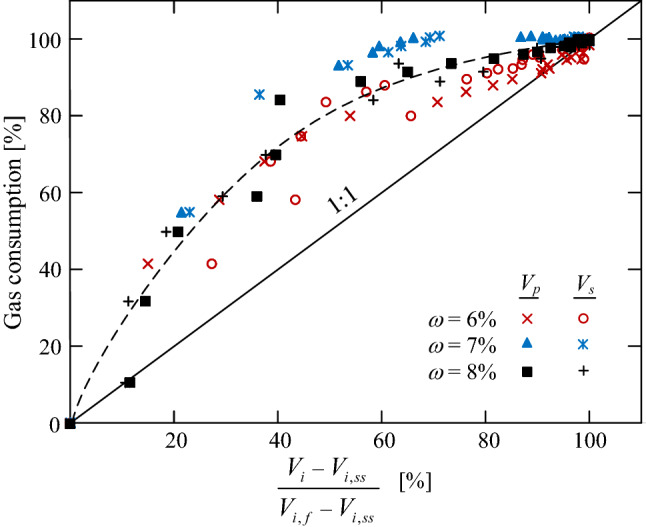
The net increase in the P and S wave velocities versus relative gas consumption (relative to the final consumed gas volume).

The convergence of the hydrate formation may be also reflected by ultrasonic measurements throughout the formation process. Figure 5 shows the net increase in the P and S wave velocities as a function of the relative gas consumption (relative to the final consumed gas volume in each test). The net increase in the ultrasonic wave velocities is calculated as the relative increase from of the range between the initial, soil-skeleton, response ($V_i,ss$), and the final converged, MHBS, response ($V_i,f$), where *i* refers either to P or S waves. It can be inferred that the ultrasonic wave response and the gas consumption are simultaneously converged. The ultrasonic response should only be considered as complementary evidence when reviewing the convergence criterion, due to the nonlinear relationship between the gas consumption and the ultrasonic response (Fig. 5).

## Hydrate saturation calculation

The effect of the hydrate amount on the mechanical behavior of MHBS has been quantified in terms of the hydrate pore-space occupancy. The very common parameter associated with the MHBS mechanical proprieties is hydrate saturation, $S_h$, which is the ratio between the hydrate volume, $V_h$, and the void volume, $V_v$, in the sample:

1 $$\begin{aligned} S_{h}=\frac{V_h}{V_v}=\frac{W_h/\gamma _h}{V_{st}-W_s/(G_s\gamma _w)} \end{aligned}$$

where $\gamma _w$ is the water density, $\gamma _h$ is the methane hydrate density, $V_{st}$ is the sample total volume, $W_s$ is the weight of soil solids, $G_s$ is the solid specific gravity, and $W_h$ is the weight of hydrate in the sample (which cannot be directly measured). $W_h$ has been evaluated in various works using different methods; either as a function of the initial water content^10,27^, the amount of consumed gas during the formation process, the amount of released gas during the dissociation process^5^, or through correlation with eclectic measured bulk resistivity^28^. This section presents three methods for $W_h$ calculation, followed by a test results comparison. For convenience, all related parameters for the calculations shown below are summarized in Table 1.

**Figure 6:**
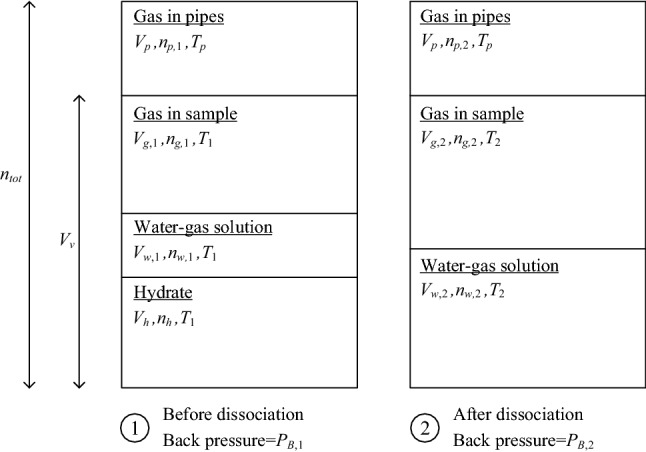
The backpressure system volumes, before and after the undrained dissociation.

1. **Theoretical full water conversion -** $W_{h,1}$
  This calculation is based on the assumption that all available water in the soil sample are converted into hydrate. Under this assumption, the ratio between $W_{h,1}$ (i.e., $W_{h}$ in this method) and $W_w$ can be directly related to the stoichiometric ratio of the methane hydrate structure, *N*, given by:
  2 $$\begin{aligned} \frac{W_{h,1}}{W_{w}}=\frac{Nm_{w}+m_{m}}{N m_{w}} \end{aligned}$$
  where $m_w$ and $m_m$ are the molar masses of water (18.02 g/mol) and methane (16.04 g/mol). This calculation ignores the fact that the initial water content may not be entirely converted into hydrate in the excess-gas method.
2. **Consumed gas method—**$W_{h,2}$
  The hydrate weight calculated in this method ($W_{h,2}$) relies on the conversion of the methane consumed by the hydrate during the formation process. Instead of accounting for the gas in the entire $P_B$ system (the soil pore-space and the $P_B$ lines), in this method, the hydrate amount is calculated based on measuring only gas-volume changes during the hydrate formation; $\Delta V_{gfh}$ (i.e., gas for hydrate). The gas-expansion effect (which is not related to hydrate formation) is reduced by:
  3 $$\begin{aligned} \Delta V_{gfh}=\Delta V_{g} - \alpha V_{g,bp} \Delta T \end{aligned}$$
  where $\alpha$ is the methane-gas thermal expansion coefficient, $V_{g,bp}$ is the gas volume in the $P_B$ system, and $\Delta T$ is the temperature change, in which the product $\alpha V_{g,bp}$ can be explicitly extracted from the gas-volume change response during the cooling process (before hydrate formation; Figure 2). $\Delta V_{gfh}$ is used to determine the number of methane moles encapsulated in the hydrate, $n_{h}$, using the ideal gas law. Each molar unit of methane-hydrate, $\hbox {CH}_4\cdot N$ $\hbox {H}_2$O, includes one mole of methane, which therefore the number of methane-hydrate moles are given by:
  4 $$\begin{aligned} n_{h}= \Delta V_{gfh}\frac{P_B}{R T} \end{aligned}$$
  where *R* is the ideal gas constant, and *T* is temperature in Kelvin units. Therefore, the hydrate weight in this method is given by:
  5 $$\begin{aligned} \begin{aligned} W_{h,2}&=n_{h}(m_{m}+Nm_{w})\\&=(\Delta V_{g} - \alpha V_{g,bp} \Delta T)\frac{P_B}{R T}(m_{m}+Nm_{w}) \end{aligned} \end{aligned}$$
3. **Dissociated gas method -** $W_{h,3}$
  The hydrate weight calculated in this method ($W_{h,3}$) is based on the amount of methane gas released during a closed-system dissociation process. In a closed-system dissociation, the volume of the back-pressure system is kept constant while thermodynamic changes are applied. To avoid the effect of volume change in the sample, the dissociation is performed by increasing the temperature while keeping a constant effective pressure, (=CP-$P_B$).
  An illustration of the back-pressure system, which includes the sample voids and the piping system, is schematically shown in Fig. 6. Before dissociation, marked by phase #1, the gas in the $P_B$ system includes the gas within the pipes, free gas in the sample, dissolved gas in water, and gas in hydrate, in which the sample temperature is $T_1$ and the pipe-system temperature is $T_p$. After dissociation, marked by phase #2, the $P_B$ system includes the gas in the pipes, free gas in the sample, and gas-dissolved water, in which the sample temperature is $T_2$ and the pipe-system temperature remains $T_p$. the notations *V* and *n* refer to the volume and the number of methane moles in each component, respectively.
  $W_h,3$ is calculated considering isochoric (constant volume) conditions of the entire system. The volume conservation is given by:
  6 $$\begin{aligned} V_v=V_{g,1}+V_{w,1}+V_h=V_{g,2}+V_{w,2} \end{aligned}$$
  where $V_v$ is the sample voids volume, $V_{g,1}$ and $V_{g,2}$, and $V_{w,1}$ and $V_{w,2}$ are sample gas and water volumes, before and after dissociation, respectively, and $V_h$ is hydrate volume. Note, that the volume of the connected pipes, $V_p$, is constant, which is therefore omitted in Eq. 6. However, the number of gas moles in the pipes system is changed due to the back pressure increase during the dissociating. Therefore, the conservation of methane moles is given by:
  7 $$\begin{aligned} n_{p,1}+n_{g,1}+n_{w,1}+n_h=n_{p,2}+n_{g,2}+n_{w,2} \end{aligned}$$
  where $n_{p,1}$ and $n_{p,2}$, $n_{g,1}$ and $n_{g,2}$, and $n_{w,1}$ and $n_{w,2}$ are the number of methane moles in the pipes, free-gas and water-solution, before and after dissociation, respectively. The conservation of water moles in the system is given by:
  8 $$\begin{aligned} n_h N=\frac{\gamma _w}{m_w}(V_{w2}-V_{w1}) \end{aligned}$$
  The number of methane moles in each substance (gas, dissolved-water and hydrate) is calculated by:
  9a $$\begin{aligned} n_{g,i}&= \frac{P_{B,i} V_{g,i}}{R T_i} \end{aligned}$$
  9b $$\begin{aligned} n_{w,i}&= \frac{\chi \gamma _w V_{w,i}}{m_m}\end{aligned}$$
  9c $$\begin{aligned} n_h&= \frac{V_h \gamma _h}{N m_h} \end{aligned}$$
  where $m_h$ is the molar wight of hydrate (=$m_m+N m_w$), and $\chi$ is solubility of methane in water (under the $P_B$ thermodynamic conditions). Finally, *W*_*h*__,3_ can be expressed by:
  10 $$\begin{aligned} \begin{aligned} W_{h,3}&=m_h n_{h}\\&=m_h \frac{P_{B,2}\frac{T_1}{T_2}\left( V_v- V_{w,2}+\frac{T_2}{T_P} V_p\right) -P_{B,1}\left( V_v-V_{w,2}+\frac{T_1}{T_P} V_p\right) }{ P_{B,1}\left( N\frac{m_w}{\gamma _w} - \frac{m_h}{\gamma _h}\right) + RT_1\left( 1-\frac{m_w}{m_m} N \chi \right) } \end{aligned} \end{aligned}$$
  where the sample void volume is given by:
  11 $$\begin{aligned} V_v=V_{st}-\frac{W_s}{\gamma _w G_s} \end{aligned}$$
  where $G_s$ is the solid specific ratio ($\sim$2.65 for quartz sand), while the sample total volume, $V_{st}$, and the solid weight, $W_s$, are laboratory measured quantities. The remaining water after dissociation, $V_{w,2}$, is the same water volume measured at the sample assembling stage, given by:
  12 $$\begin{aligned} V_{w,2}=\frac{W_t \omega }{\gamma _w} \end{aligned}$$
  where the total sample weight, $W_t$, and the initial water content, $\omega$, are measured quantities.
  As an alternative to the closed-system dissociation, one can estimate the hydrate amount in the sample by measuring the released methane gas volume from the system either by designated flow-meters or by gas collectors.

**Table 1:** Parameters used to calculate the hydrate saturation (Eqs. 1-12).

Description	Symbol
Hydrate saturation	$S_{h}$
Volume of hydrate	$V_{h}$
Sample void volume	$V_{v}$
Sample total volume	$V_{st}$
Gas volume	$V_{g}$
Gas-dissolved water volume	$V_{w}$
Volume of the back pressure pipes	$V_{p}$
Gas volume consumed by hydrate	$V_{gfh}$
Gas volume in the back-pressure system	$V_{g,bp}$
Methane hydrate density	$\gamma _{h}$
Water density	$\gamma _{w}$
Solid specific gravity	$G_{s}$
Weight of solid in the sample	$W_{s}$
Weight of hydrate	$W_{h}$
Weight of water	$W_{w}$
Solubility of methane in water	$\chi$
Methane hydrate stoichiometric number	*N*
Molar mass of water	$m_{w}$
Molar mass of methane	$m_{m}$
Molar mass of hydrate	$m_{h}$
Number of methane moles in the hydrate	$n_{h}$
Number of methane moles in the water solution	$n_{w}$
Number of methane moles in the methane gas	$n_{g}$
Number of methane moles in the pipes system	$n_{p}$
Sample temperature before hydrate dissociation	$T_{1}$
Sample temperature after hydrate dissociation	$T_{2}$
Temperature in the pipes system	$T_{p}$
Pore fluid pressure (back pressure)	$P_{B}$
Ideal gas constant	*R*
Thermal expansion coefficient of methane gas	$\alpha$

**Figure 7:**
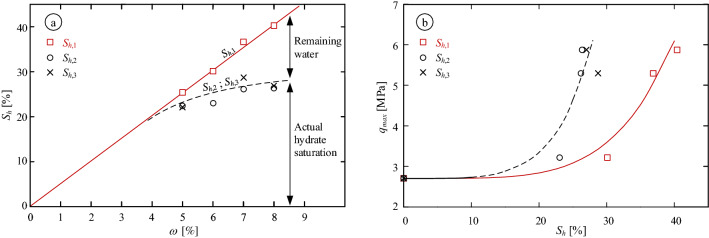
(**a**) Comparison between the three $S_h$ calculation methods as a function of the initial water content, $\omega$, and (**b**) the maximum deviatoric stress, $q_{max}$, as a function of three calculated $S_h$.

Figure 7a presents a comparison between the three $S_h$ calculation methods for MHBS formed by different initial water contents. Values of $N = 5.75$, $\gamma _h = 0.913 \,\hbox {g/cm}^3$ and $\chi =0.037 g/kg$ were considered. As can be seen, $S_{h,1}$ follows a linear trend, in accordance with the theoretical assumption that all the water are converted into hydrate, while $S_{h,2}$ and $S_{h,3}$ show lower values. $S_{h,2}$ and $S_{h,3}$ follow similar trends as they are both calculated based on the net gas consumed for hydrate crystallization (i.e., actual hydrate saturation, as indicated in the figure). The difference between the two trend lines reflects the effect of the remaining water, which was not converted into hydrate, even though the hydrate formation was converged. Figure 7b shows the maximum deviatoric stress, $q_{max}$ obtained in the triaxial tests. Two distinct trends can be drawn for the water conversion calculation, $S_{h,1}$,and the gas conversion calculation, $S_{h,2}$ and $S_{h,3}$ (for convenience, power-law shape-function was adjusted). As can be seen, $q_{max}$ values may be associated with quite different $S_h$ values, which may yield uncertainty in characterizing the mechanical behavior of MHBS.

The sensitivity of the assumed properties on the calculated hydrate saturation values needs to be examined. For example, the commonly assumed stoichiometric number for methane hydrate is $N=5.75$, which corresponds to full hydration^36^. However, a value of $N=6$ may be considered for average hydration, for which a deviation of about $-1\%$ is obtained for $S_{h1}$ values and 4% for $S_{h2}$ and $S_{h3}$ values. Another parameter for the sensitivity examination is the solubility of methane in water, which was assumed to be $\chi =0.037 \,\hbox {g/kg}$. It should be noted that for methane hydrate, this parameter may be negligible, yielding no change in $S_{h1}$ and $S_{h2}$ and up to 0.3% deviation in $S_{h3}$. Note, that for $\hbox {CO}_2$ hydrate-bearing sediments, the (relative high) water-solubility should not be ignored in the calculation of $S_{h3}$, as it reduces the $S_{h3}$ value by approximately 3.5% (with comparison to $\chi =0$). One should also consider the effect of the laboratory measurements on the $S_h$ values. For example, inaccuracy in the gas pump temperature measurements (which is not standardly taken) may yield a deviation of about 0.3% per $1 \, ^{\circ }\hbox {C}$ temperature inaccuracy (depending on the experimental setup).

## Homogeneous sample preparation

Hydrate is a solid that characterized by high stiffness and strength properties^42^, which its occupancy in the soil pore space dramatically affect the overall mechanical behavior. The hydrate mechanical effect depends on the hydrate saturation, $S_h$, the pore-scale morphology, and the hydrate distribution in the porous. Thus, the homogeneity of the water/ice (which will be later converted into hydrate) during the sample assembling may affect the mechanical response of the produced MHBS sample. A thorough study regarding the hydrate distribution in the pore space is presented by Kneafsey et al.^22^ and Lei et al.^24,25^. These works show that achieving a perfectly homogeneous hydrate distribution is extremely challenging, even if the sample has a homogeneous (initial) pore water distribution. However, a heterogeneous initial water distribution significantly reduces the hydrate homogeneity in the sample. The excess-gas method enables us to control the initial water distribution during the sample assembling stage, and therefore the mixing method used to produce homogeneous water may be crucial for the formation of a homogeneous MHBS. Note, that the homogeneity described above refers to a macro-scale (first order) homogeneity, in oppose to micro-scale hydrate non-homogeneity evidence, presented in CT and X-ray studies^22,24,25^.

**Figure 8:**
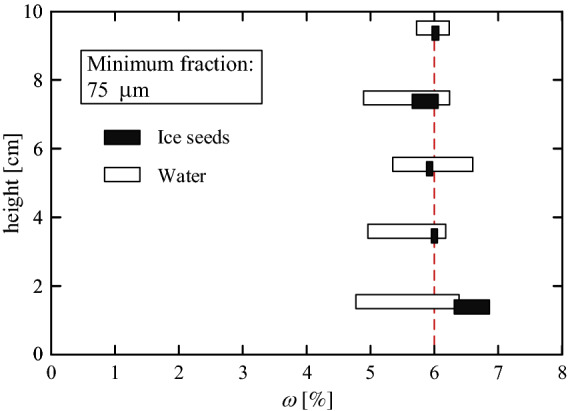
Horizontal distribution of water content at different heights in a soil sample which was initially mixed with a global amount of $\omega =6\%$.

The partial saturation state of a soil sample can be achieved by various methods. For example, Miyazaki et al.^26^ fully saturated a sand sample and then reduced the water content by draining the sample using a syringe pump. Other works^5,7,27^ mixed sand with a predetermined water content. However, creating a homogeneous mixture of solids (soil grains) and liquids (water) is challenging. The homogeneity can be improved by mixing frozen sand and ice seeds, where a mix of solids yields a more homogeneous product (even if it will be later defrosted)^7,10^. The advantage of using ice-powder mixture rather than water moisture is demonstrated in Fig. 8. The figure shows an example of internal water content distribution, $\omega$, in the vertical and horizontal directions, of a soil sample which initially mixed with a global amount of $\omega =6\%$. Although the same mixing procedure was utilized, the figure shows that the water mixture yields a sample with up to 20% horizontal deviation from the designed $\omega$ value, while the ice-seed mixture yields a much smaller deviation.

**Figure 9:**
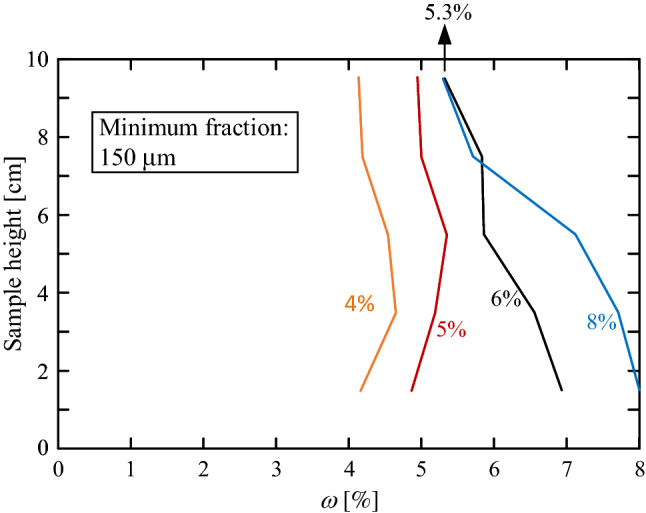
Vertical $\omega$ distributions of samples with different initial water contents.

While the horizontal $\omega$ distribution indicates a quality of mixing, the vertical distribution may indicate soil-skeleton properties associated with the capacity of capillary water. In other words, the relative uniform vertical distribution shown in Fig. 8 was achieved as a result of the relatively low water content used in that specific sand sample. Figure 9 shows vertical $\omega$ distributions of different initial water contents. As can be seen, low initial water content yields fare uniform distribution (4 and 5% in the figure), while high initial water content (6 and 8%) yields non-uniform distribution. In addition, while the top measuring point of the $\omega = 4$ and 5% samples increases in accordance to the applied global water content, the top $\omega$ values of both the 6 and 8% samples are similar ($\sim 5.3\%$). This $\omega$ capillary threshold may be considered as a soil property, associated with the soil skeleton specific surface area.

**Figure 10:**
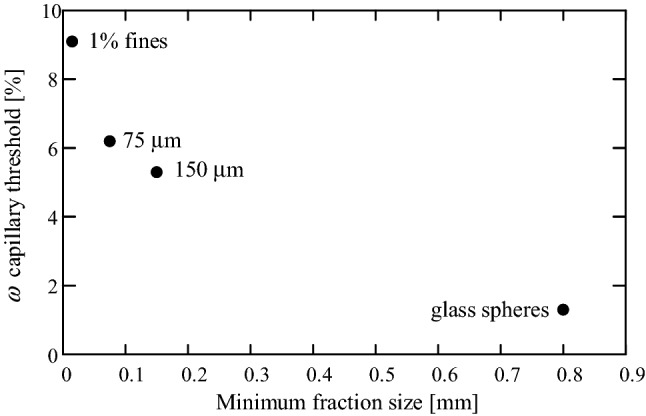
Water capillary thresholds for four examined grain assemblies; three different grading of the same soil and a glass spheres assembly.

Note, that the $\omega = 6\%$ distributions in Figs. 8 and 9 are different, because they were measured in soils with different minimal fraction sizes (75 and $150 \,\mu \hbox {m}$, respectively). Figure 10 shows measured water capillary thresholds of four examined grain assemblies; three different gradings of the same soil and a glass spheres assembly. As can be seen, soil samples with smaller fractions are associated with higher water capillary threshold.

Therefore, to ensure a first-order (macro-scale) homogeneous hydrate distribution within the host soil, using the excess-gas method, it is important to assess the capillary threshold of the examined soil and to examine the quality of the water-soil mixing.

## Water saturation

In the testing procedure, after the hydrate formation, one can saturate the MHBS with water (replacing the remaining gas) for simulating the marine environment. Hyodo et al.^21^ examined the mechanical difference between water-saturated and gas-saturated MHBS in triaxial testing. The gas-saturated MHBS showed much higher strength and stiffness responses than the water-saturated samples. Pinkert^43^ showed that the fundamental mechanical difference between the two cases is because gas-saturated MHBS exhibits cohesion trends while the water-saturated MHBS does not show true–cohesion trends. An optional explanation for this mechanical difference was suggested, in which the water solution dissolves hydrate-hydrate cementation bridges. Therefore, to allow a comparative study between various test results, one should strictly monitor and report the mechanical changes during the saturation process, such as: (1) the used water solution, as one can use distilled water, saline water, or gas-dissolved water, (2) whether hydrate formation/dissociation or volume change develops during the saturation process, and (3) other arisen technical issues, such as flow blockages throughout the process along with the method used to overcome them.

## Discussion

**Figure 11:**
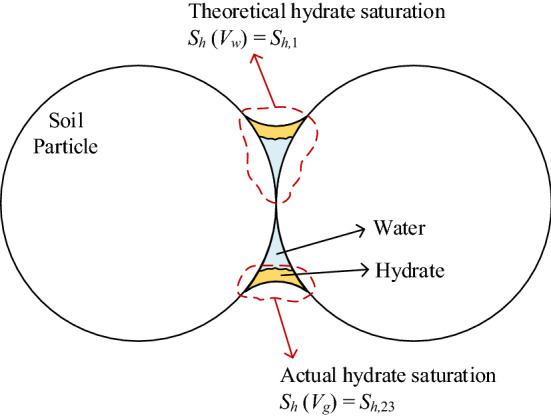
An illustrative difference between the theoretical and the actual $S_h$.

This work shows experimental examples of actual (measured) versus theoretical hydrate saturations that may be deduced in the excess-gas hydrate-formation method. In the excess-gas formation method, the hydrate is formed at the capillary water between grain contacts, from the water-gas interface and inward the water droplets, in which not all the water is necessarily converted into hydrate. Therefore, besides the actual formed hydrate saturation (in quantity terms), the mechanical behavior may be affected by the geometrical arrangement of the hydrate, which is governed by the initial water content arrangement. Figure 11 illustrates the schematic difference between the theoretical and the actual $S_h$, with relation to the three $S_h$ calculation methods. In parallel to the investigation of structural elements that are studied both by their section area (reflecting material quantity) and their moment of inertia (reflecting the geometrical arrangement), the hydrate effect in MHBS should be studied by the mutual effect of both the actual $S_h$ and its geometrical arrangement (related to the initial water content). In other words, the effective hydrate saturation, $S_{h,eff}$, which should be used for mechanical correlations, may be evaluated in the range between $S_{h,2}\approx S_{h,3}$ ($\equiv S_{h,23}$) and $S_{h,1}$:

13 $$\begin{aligned} S_{h,23} \le S_{h,eff}\le S_{h,1} \end{aligned}$$

As a laboratory MHBS formation aims to mimic the mechanical behavior of natural MHBS, one should adjust the testing conditions to the desired mechanical product. For example, for the simulation of samples involving numerous hydrate-hydrate cementation bridges within the pore-space, the gas-saturation method may be preferred over the water-saturation method, to avoid the laboratory effect of hydrate-bridges dissociation during the water saturation process (even though natural MHBS is typically underwater). In that case, the effective hydrate saturation, $S_{h,eff}$, should be well defined.

Each of the laboratory hydrate formation methods involves various fundamental and technical advantages and disadvantages. The excess-gas method has the advantage of a relatively short testing duration (about a few days in total). In addition, the testing procedure in this method is relatively simple (with comparison to other methods), such that it enables a relatively repeatable MHBS formation, which increases the testing validity and efficiency. The major disadvantage of the excess-gas method is that it does not necessarily mimic the natural hydrate morphology, which is most probably associated with a pore-filling hydrate than grain-contacts hydrate morphology. A reliable data analysis could overcome this disadvantage by allowing the correlation between laboratory experimental results and engineering simulations of natural MHBS (through the definition of $S_{h,eff}$). From the experimental aspect, one should report all testing details, such as $S_h$ calculation, detailed water saturation process, homogeneity assurance, host-soil grading, etc. Such detailed reporting will enable us to accurately compare different data sets or to combine results in a unified analysis.

## Conclusions

This paper presents an experimental study of aspects that influence the geotechnical investigation of laboratory formed MHBS using the excess-gas method. There are uncertainties or inconsistencies regarding some of the testing procedures among the different works, which limits the comparison and inclusion of different experimental data sets. We propose a set of experimental recommendations for geotechnical testing based on the excess-gas formation method, that will serve as a basis for integrating various data-sets:

- *Sample homogeneity*, in which it is suggested to ensure a homogeneous moisture content (which will be later converted into hydrate) in the sample preparation stage. An ice-powder mixture was found to be more efficient in producing a homogeneous partially saturated sample (over water mixture). In addition, one should determine the host-soil capillary threshold to avoid non-homogeneous gravimetric water distribution.
- *Temperature measurement*, in which in geotechnical testing, the temperature is typically measured outside the examined sample (to avoid the mechanical effect of the thermometer). The effect of temperature difference between the confining liquid and the sample should be considered in temperature change procedures, such as hydrate formation or dissociation. In the hydrate formation process, the temperature difference can affect the determination of the formation duration (as the cell liquid may reach the hydrate phase boundary before the sample). In the investigation of dissociation processes, one should perform a preliminary estimation of an apparent phase boundary that corresponds to the measured temperature in the test apparatus.
- *Formation duration*, in which it is recommended that one should clarify the chosen criteria for the formation start- and end-points, along with reporting the hydrate formation duration.
- *Hydrate saturation calculation*, in which the $S_h$ calculation scheme has to be reported. It is recommended to report both the initial water content (or the maximum theoretical $S_h$) in addition to the hydrate saturation that is evaluated from the gas amount consumed by the hydrate. The effective hydrate saturation (for mechanical investigation) should be studied in terms of both calculation products, as the first may be related to the geometrical arrangement of the hydrate in the porous and the second is related to the actual formed hydrate quantity.
